# Studies in the Psychology of Sex

**Published:** 1902-05

**Authors:** 


STUDIES IN THE PSYCHOLOGY OF SEX. Sexual Inversion. By HAVELOCK ELLIS, L. S. A. (England). Fellow of Medico-Legal Society of New York and the Anthropological Society of Berlin; Honorary Fellow of the Chicago Academy of Medicine, etc.; General Editor of the Contemporary Science since 1899. Volume II. Completed in five volumes. Pages xi.—272. Philadelphia: F. A. Davis Co. [Extra cloth, $2.00 net, delivered.]

The thoroughness with which the author has written will be admitted by all who read the book. The value of his researches will be questioned by many.

The German dictum, "nichts neues," will be pronounced by those who require some advance in a special field of knowledge as an excuse for a new book. Indecent and unnecessary will be the judgment of many.

Our own opinion of the volume before us is that it presents in an interesting and compact form the existing knowledge on this subject. Historically considered, it is very complete; psychologically it is interesting and valuable because of the confessions which are made by men and women inverts.

The book must be judged as to its usefulness. We can see little good accomplished for, in the author's words, "we can seldom safely congratulate ourselves on the success of the cure of inversion."

Those who desire to study the subject of sexual inversion will find this book a most useful one. J. W. P.

